# The sink as a potential source of transmission of carbapenemase-producing *Enterobacteriaceae* in the intensive care unit

**DOI:** 10.1186/s13756-017-0182-3

**Published:** 2017-02-16

**Authors:** Deborah De Geyter, Lieve Blommaert, Nicole Verbraeken, Mark Sevenois, Luc Huyghens, Helena Martini, Lieve Covens, Denis Piérard, Ingrid Wybo

**Affiliations:** 1 Department of Microbiology, Vrije Universiteit Brussel (VUB), Universitair Ziekenhuis Brussel (UZ Brussel), Laarbeeklaan 101, 1090 Brussels, Belgium; 2 Department of Intensive Care Unit, Vrije Universiteit Brussel (VUB), Universitair Ziekenhuis Brussel (UZ Brussel), Laarbeeklaan 101, 1090 Brussels, Belgium

**Keywords:** Carbapenemase-producing *Enterobacteriaceae*, Hospital sinks, Outbreak, Intensive care unit, *Citrobacter freundii* OXA-48, Transmission

## Abstract

**Background:**

Carbapenemase-producing *Enterobacteriaceae* (CPE) are emerging pathogens that represent a major public health threat. In the University Hospital of Brussels, the incidence of new patients with CPE rose from 1 case in 2010 to 35 cases in 2015. Between January and August 2015, five patients became infected/colonized with CPE during their stay in the same room in the intensive care unit (ICU). Since the time period between those patients was relatively short and the strains belonged to different species with different antibiograms and mechanisms of resistance, the hypothesis was that the environment could be a possible source of transmission.

**Methods and results:**

Environmental samples suggested that a contaminated sink was the source of the outbreak. Besides other strains, *Citrobacter freundii* type OXA-48 was frequently isolated from patients and sinks. To investigate the phylogenetic relationschip between those strains, pulsed-field gel electrophoresis was performed. The strains isolated from patients and the sink in the implicated room were highly related and pointed to sink-to-patient transmission. In total, 7 of 8 sinks in the isolation rooms of the ICU were found to be CPE contaminated. To control the outbreak, the sinks and their plumbings were replaced by new ones with another structure, they were flushed every morning with a glucoprotamin solution and routines regarding sink practices were improved leading to discontinuation of the outbreak.

**Conclusions:**

This outbreak highlights that hospital sink drains can accumulate strains with resistance genes and become a potential source of CPE.

## Background

Carbapenemase-producing *Enterobacteriaceae* (CPE) represent a major public threat in both the acute and chronic care sectors as well as in the community. Treatment of patients infected by CPE is a challenge since only a few drugs remain active against these strains [1]. Between 2010 and 2014, our healthcare facility was confronted with a rising number of CPE positive patients, with 26 confirmed cases by 2014. Patients in the intensive care unit (ICU) comprised the majority of all colonized or infected patients with CPE. Despite screening and enhanced cleaning, the incidence rose further in the first half of 2015 and could be linked to contaminated sinks.

It is described that sink drains beneath washbasins in hospitals contain 10^6^–10^10^ colony forming units (CFU)/ml of bacteria of which approximately 10^3^–10^5^ CFU/ml are Gram-negative (GN) rods, especially waterborne bacteria [2]. These bacteria can colonize/infect the patient via different transmission routes. Contaminated sinks have been implicated in several outbreaks. Kramer and colleagues described that sinks can be hidden reservoirs generating large quantities of aerosols. They found that 100% of the sinks in a neonatal ICU were contaminated with GN rods [3]. Starlander and co-workers noticed that four patients became infected or colonized by an ESBL producing *Klebsiella pneumoniae* strain during a seven month period on the neurological ICU. Environmental sampling led to the conclusion that the plughole of the sink was the source of transmission [4]. The group of Lowe described an outbreak with an ESBL producing *Klebsiella oxytoca*. Cultures from hand washing sinks in the ICU unit yielded *Klebsiella oxytoca* with identical molecular patterns to cultures from the patients [5]. Wolf and colleagues discovered that mechanically ventilated patients were sometimes colonized with bacteria positive for ESBLs. During a five month observation period, four patients became colonized with ESBL positive bacteria that were genetically identical to those that had previously been isolated from the sink [6]. Roux and co-workers found that 31% of the 185 sinks in their ICU were contaminated with ESBL positive *Klebsiella* and *Enterobacte*r species [7]. The group of Leitner published an article about contaminated handwashing sinks as the source of a clonal outbreak with a resistant *Klebsiella oxytoca* strain on a hematology ward [8].

The aim of this study was to describe an outbreak of CPE in the ICUs of the UZ Brussels and to investigate whether the sink could be a possible source of transmission and in addition, whether these transmissions could be avoided in the future by means of improving infection control measures and replacing the sinks by new ones. This study highlights the fact that sink drains can be hidden reservoirs for CPE which is in general not known by health care workers and even by infection control specialists.

## Methods

### Setting

The University Hospital Brussels is a teaching hospital with more than 700 beds. There are four ICUs for adults. Each unit contains six beds, two of them are placed in a single room that can be closed (room one and six; room one has an anteroom). These two separate rooms are predominantly in use for patients who need additional isolation precautions. Every unit has eight sinks: one for every patient, one in the anteroom and one placed centrally. In total there are thus 32 sinks available in the ICUs.

### Case definitions

New cases of patients with CPE were defined as patients (infected or colonized) identified in the ICUs in 2015 (not known with a CPE before 2015 or not known in another hospital) with a CPE positive culture from any site. Development of a colonization/infection with CPE means that rectal screening samples were negative on admission. An outbreak can be defined as at least two cases linked by an epidemiological chain of transmission.

### CPE surveillance and isolation policy

Patients in the ICU are screened rectally on admission; on discharge and weekly for all patients hospitalized more than one week. When a screening is considered as positive, the patient is isolated on contact precautions in a single room with use of gloves and a disposable overcoat.

### Environmental cleaning policy

The room has to be cleaned daily with Incidin® Plus (0.5% glucoprotamin). At discharge, the room is cleaned intensively and equipment such as gloves and hand alcohol are discarded. Periodic checks of the quality of terminal cleaning are performed with the Glowcheck® (Hartmann, Heidenheim, Germany).

### Microbiological methods

#### Environmental sampling and microbiological methods

Swabs (eSwab, Copan, Brescia, Italy) from the plughole of the sink (10–15 cm depth), the environment of the sink and room (high-touch surfaces such as the bed, monitor, door knob, the Velcro of the blood pressure device, matrass and curtain) were taken at several time points. After sampling, 2 ml of Fastidious Organisms Broth (FB, own preparation) was added to the eSwab and incubated for 24 h on 35 °C. After one day, sink and environmental specimens were investigated for the presence of CPE on two separate chromogenic media: chromID® CARBA and OXA-48 (bioMérieux, Marcy l’Etoile, France) 48 h after sampling (24 h incubation on 35 °C). Identification of suspicious colonies was performed by matrix-assisted laser desorption ionization–time of flight mass spectrometry (MALDI-TOF MS) using a Microflex LT mass spectrometer with MALDI Biotyper 3.0 software and Reference Library 3.2.1.0 (Bruker DaltonikGmbH, Bremen, Germany).

Antibiotic susceptibility testing was performed by the disk diffusion method and by using the interpretative criteria of The European Committee on Antimicrobial Susceptibility Testing (EUCAST) combined with recommendations of the National Reference Center (NRC) and BAPCOC (Belgian Commission for the Coordination of the Antibiotic Policy). Colonies considered as suspicious for the production of a carbapenemase need further molecular characterization as a confirmation step.

#### Air sampling experiments

The bacterial aerosol was measured 10 cm above the sinks during tap water running over 10 min and compared with results without running tap water. The measurements were performed with the MAS-100® Airsampler (Merck Millipore, Darmstadt, Germany); programmed to measure 100 L air per minute and containing MacConkey plates (bioMérieux, Marcy l’Etoile, France) specific for the detection of GN bacteria. These plates were incubated for 18–24 h on 35 °C. Samples from the environment of the sink were also taken. Further identification and susceptibility testing was performed as described previously (in “Environmental sampling and microbiological methods”).

#### Molecular characterization

A) Bacterial colonies suspicious for the production of carbapenemases
  Molecular characterization was performed using the Xpert Carba-R Assay on the GeneXpert® system (Cepheid, Sunnyvale, California, USA). Colonies considered as suspicious for the production of carbapenemases but with a negative result on the GeneXpert®, were send to the NRC of CPE to exclude resistance.
B) Genetic relatedness
  To confirm genetic relatedness between isolates from patients and environmental samples, pulsed-field gel electrophoresis (PFGE) was performed.

#### Patients’ samples

Sampling in patients is part of routine daily practice. Screening samples are taken via rectal swabs (eSwab, Copan, Brescia, Italy). For rectal screenings and other clinical samples, the same protocol as for environmental sampling is followed, except that they are not enriched with FB.

## Results

### Epidemiological investigation

Between 2010 and 2015, the number of new patients with a CPE per year on the ICU rose from 1 to 21 and the most abundant type became the *Klebsiella pneumoniae* carbapenemase. In 2015, patients with CPE staying on the ICU contributed 67% of all cases with CPE. Between January and August 2015, five patients whose initial screenings were negative, developed CPE carriage/infection during their hospital stay on the ICU A in bed 6. In Table 1, the isolates from the five patients who became positive for CPE on ICU A, room 6 are represented. The isolates belonged to different species with different antibiograms. The time period between the detection of these CPE positive patients was relatively small and since carbapenemase resistance determinants are located on genetic mobile elements, the environment was suspected as a possible source of transmission.

**Table 1:** CPE carriage/infection on the ICU A, bed 6

Patient 1 (colonization)	KPC, OXA-48 and NDM (2/2/2015). Different species
Patient 2 (infection site: endotracheal aspirate)	KPC, OXA-48 and NDM (23/2/2015). Different species
Patient 3 (colonization)	OXA-48 and NDM (18/5/2015). Different species
Patient 4 (infection site: abdominal abscess)	OXA-48 (15/7/2015). Different species
Patient 5 (infection site: endotracheal aspirate)	NDM and OXA-48 (30/8/2015). Different species

Five patients developed CPE carriage/infection during their hospital stay on the ICU A, room six between January and August 2015. They all had negative screenings on admission. The isolates belonged to different species with different antibiograms. The time period between the detection of these CPE positive patients was relatively small

### Environmental investigation

Swabs on dry surfaces taken were taken as well as swabs from the sinks’ drain in room 6, ICU A. Two samples became positive for CPE: the sinks’ drain and the blood pressure cuff although a procedure for cleaning was in place for the latter. The samples from other high-touch surfaces were all negative for CPE, indicating that the cleaning was appropriate.

To investigate whether other isolation rooms were also carrying CPE in their sinks, samples were taken from the drains. Besides one, every isolation room carried CPE in the sink. Several species and types of CPE were isolated: *Klebsiella pneumoniae* NDM, *Klebsiella pneumoniae* KPC, *Klebsiella pneumoniae* OXA-48, *Enterobacter cloacae complex* OXA-48, *Citrobacter freundii* OXA-48, *Citrobacter freundii* OXA-48 + NDM, *Klebsiella oxytoca*/*Raoultella species* OXA-48, and *Escherichia coli* OXA-48, suggesting that resistance genes did accumulate in the drains as patients with CPE were preferably cared for in this rooms. The results also revealed that in some rooms, the bacterial flora in the sink was probably the same as the bacteria cultured in the patient. Between the end of July and the end of August 2015, 5 patients were positive for CPE that were probably the same as in the sinks. Three of them were not known with a CPE on admission and here it can be stated that the sink colonized/infected the patients. From those three patients, two of them had positive respiratory samples with CPE.

**Fig. 1:**
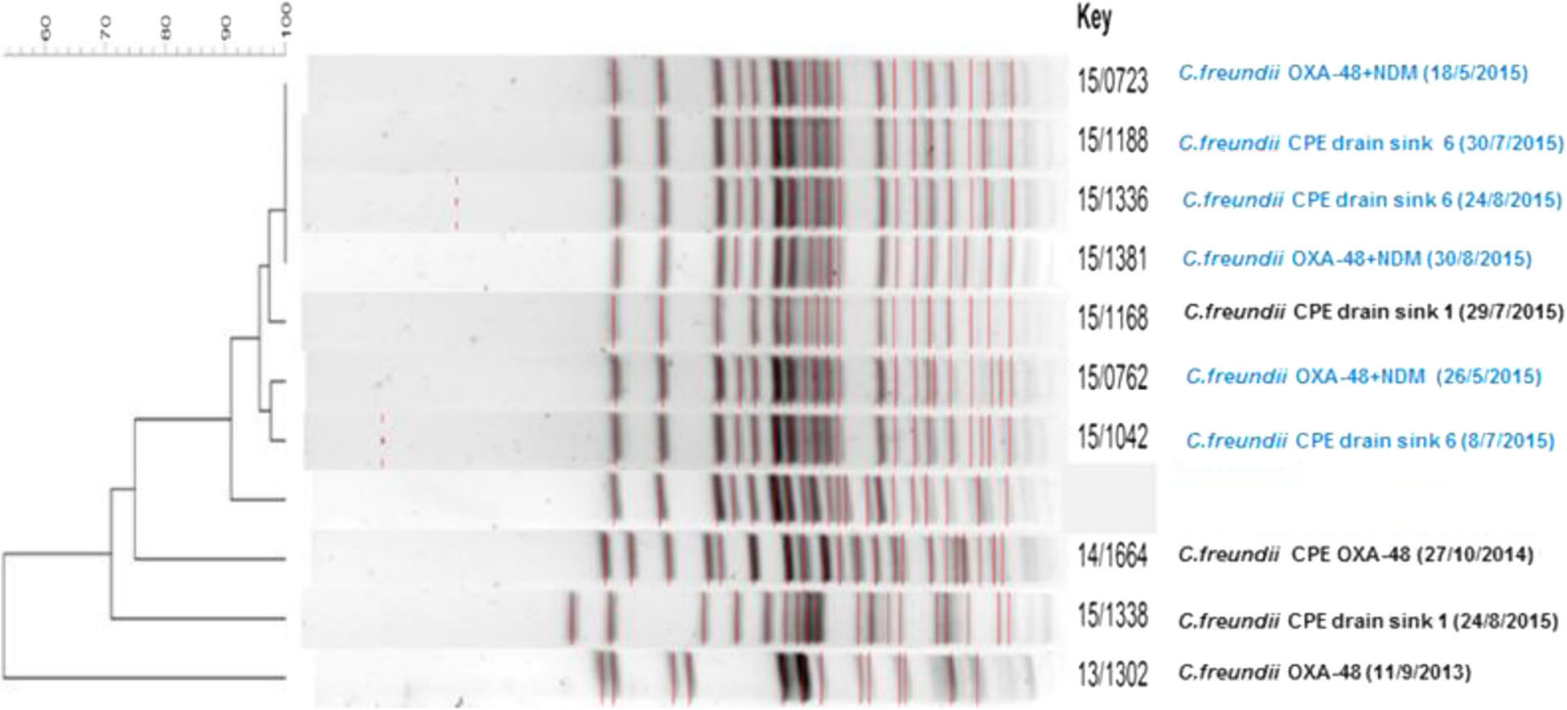
Phylogenetic relatedness between *Citrobacter freundii* CPE strains. On the *left* side of the figure, a dendogram is shown representing the relationship between the strains in %. In *blue*, the patients’ and drain cultures from ICU A bed 6 are shown and in *black*, the patients’ and drain cultures from ICU A bed 1are represented together with the date of isolation. The strains isolated from patients and sinks in room 6 are highly related

In 2015, *Citrobacter freundii* type OXA-48 was frequently isolated from patients and sinks. To investigate the relatedness between those strains, PGFE was performed. *Citrobacter freundii* CPE positive strains from patients staying on the ICU isolated in 2013 and 2014 were also included. These results showed that the strains isolated from patients and sinks in room 6 are highly related (Fig. 1).

### Infection control measures

In the weeks and months following the previous results, the infection control team tried to get rid of the outbreak by implementing new infection control measures in addition to existing ones. Medical staff, nurses and the cleaning personnel were informed about the outbreak, presentations were given, the extreme importance of correct hand hygiene was highlighted and measurements on intensively cleaning were performed. Since the blood pressure cuff yielded a CPE, it was decided to work with disposable material. There was an agreement to replace all the siphons in the isolation rooms and to culture the siphons in the other rooms. A daily disinfection of the sinks with Incidin® Plus (Microtek, Zutphen, The Netherlands) was implemented. The sinks were used for washing hands and medical instruments before disinfection but also to flush patient’s substances such as dialysis removals containing antibiotics and microorganisms. These actions can promote biofilm formation and selection of resistant bacteria. Therefore, it was decided to use the sinks only for “clean work”. For the removal of dialysis fluids, special waste containers were bought.

### Follow up

Between September and the end of December 2015, swabs of the plugholes of the sinks were taken on a regular base in every room of the ICUs. Despite all the efforts made, still 9 out of the 32 sinks (28%) were positive for CPE at the end of September 2015. During that time period, three patients had a sample showing CPE but two of them were already positive on admission. A third person had a respiratory infection with a CPE but in his case, it was not possible to know whether this infection was nosocomial or not since he was not screened on admission.

Because of the high number of siphons containing CPE even after replacement, the technical department was asked to replace all the whole sinks (not only the siphons) by newly engineered ones with an open inlet. This makes cleaning easier and prevents the formation of biofilms.

In general: between the 1^st^ of January and the 31^st^ of December 2015, 21 patients became colonized/infected with a CPE on the ICUs; 8 of them probably due to a contaminated siphon. Since the replacement of the whole sinks at the end of 2015, only one patient became positive with a CPE in March 2016 after he had been hospitalized on the ICU. At that moment, he was already hospitalized for four weeks. The drain of the sink in the room where the patient had stayed was positive with CPE, although it was another species and strain. In this case, it was hard to say whether the CPE was transmitted from the sink to the patient or the other direction since the patient was not screened on admission. The other sinks stayed negative until the last day of the sampling and no other transmissions were noted.

Airsampling experiments with two sinks showed that it is possible that bacteria in the biofilm of the sink can get carried up into the air above the drain through aerosols when tap water is running, since we were able to pick up Gram-negative bacteria in the air and the environment on the sink after tap water was running. In the air, *Stenotrophomonas maltophilia*, *Serratia marcescens*, *Pseudomonas oleovorans* group and *Pseudomonas putida* were recovered. On the taps, *Serratia marcescens and Pseudomonas fluorescens* group were found and on the rims*, Serratia marcesens* CPE suspected and *Stenotrophomas maltophila* were cultured.

## Discussion

The prevalence of infections with multidrug resistant (MDR) GN bacteria such as CPE is increasing worldwide [9] and there is probably a wider range of environmental reservoirs for those bacteria compared with Gram positive MDR bacteria [10].

Both the results in the literature and in this work show that the sink is an ideal moist reservoir for (waterborne) GN bacteria to survive. The fact that fluids, often containing antibiotics, were flushed through the drains, promotes the selection of resistant bacterial strains. Despite the efforts made and the discontinuation of the outbreak with CPE on ICU A room six, even after replacing the complete sinks, we could find multiresistant *Pseudomonas* species and *Stenotrophomonas maltophilia*. Both bacteria can also colonize/infect patients and indeed, we noticed that some patients showed positive respiratory samples with those species after a few days of hospitalization on the ICU (data not shown). However, we didn’t do molecular characterization to prove this hypothesis.

Air sampling experiments taught us that the air above and the environment of the sink got contaminated with bacteria after tap water was running. Hands of health care workers can be contaminated via this way. This underlines the primordial importance of hand hygiene. A limitation of the study is that although we could pick-up bacteria in the air and the environment of the sink after water was running, we could not prove that these bacteria really came from the biofilm of the siphon and we were not able to pick up CPE in the air above the sink. This is possibly due to the limited time and availability of the siphons for the measurements in order to not disturb the critical ill patients in the room.

In the next years, a new ICU will be built in the UZ Brussels. A few propositions according to the architecture of the room and sinks can be made. First of all, it was considered not to place sinks in the rooms. Sinks were used to wash reusable medical devices before disinfection, as waste bins and as water suppliers for shaving men. Reprocessing of reusable medical devices should be centralized, shaving can be done by means of a separate wash basin and fluids have to be removed in special containers that provide easy transport to the utility room. Body fluids should not be flushed through the sink anymore. A second possibility is to build a room with two separate sinks. One sink should be rigorously restricted to hand washing. The sinks used for waste disposal should be systematically considered as potentially contaminated. In our ICUs, the distance between the sink and the patient’s bed is less than one meter. There are no guidelines about the minimum distance required and it depends also on the sink’s architecture, but as seen in literature, aerosols and splashes coming from the plughole of the sink can be propagated up to 1 m from the sink when the tap is turned on [11]. Therefore, we suggest that the distance between a sink and a patient’s bed should be at least two meter.

In our ICU, there are two types of taps. The taps in the isolation rooms are designed well with a distance between tap and inlet of 40 cm. In contrast, the taps in the standard rooms are not ergonomically designed: the distance between the tap and the inlet of the wash basin is too small (20 cm) which makes it possible to contaminate the tap when water is running. In the future, we will replace them. Moreover, in the sinks of the standard rooms, water from the tap is directed straight into the outlet, allowing splash-back from the sink’s drain trap.

A German company brought a self-disinfecting siphon on the market (MoveoSiphon ST24, MoveoMed, Dresden, Germany). That device prevents the formation of a biofilm in the sink by means of permanent physical disinfection, electromagnetic cleaning and antibacterial coating [12]. This siphon was tested during five months (July- November 2016) in the ICU A room 1 for the presence of GN bacteria. During that entire period, we could not pick up any GN bacteria (data not published). However; there still need to be investigated whether that siphon could really prevent nosocomial transmissions in our ICUs and whether this intervention will be cost-effective.

## Conclusions

There can be concluded that the environment is an important reservoir for MDR Gram-negative bacteria, as demonstrated in this CPE outbreak linked to contaminated sinks. Despite the efforts already made and the discontinuation of the outbreak in ICU A, some sinks are to date still contaminated with MDR strains. Therefore, different disciplines need to sit together and decide about how to change our “sink attitude” and how to make structural changes to the architecture of a patient’s room in a way that it stays both practical for the health care workers and economical.

## Abbreviations

BAPCOC: Belgian commission for the coordination of the antibiotic policy
CFU: Colony forming units
CPE: Carbapenemase-producing *Enterobacteriaceae*
ESBLs: Extended-spectrum β-lactamases
EUCAST: The European committee on antimicrobial susceptibility testing
FB: Fastidious organisms Broth
GN: Gram-negative
ICU(s): Intensive care unit(s)
MALDI-TOF MS: Matrix assisted laser desorption/ionization-time of flight mass spectrometry
MBL: Metallo-β-lactamase
MDR: Multidrug resistant
NDM: New Delhi metallo-β-lactamase
NRC: National reference center
OXA: Oxacillinase
PFGE: Pulsed-field gel electrophoresis

## References

[1] Doi Y, Paterson DL (2015). Carbapenemase-producing Enterobacteriaceae. Semin Respir Crit Care Med.

[2] Döring G, Ulrich M, Müller W, Bitzer J, Schmidt-Koenig L, Münst L, Grupp H, Wolz C, Stern M, Botzenhart K (1991). Generation of Pseudomonas aeruginosa aerosols during handwashing from contaminated sink drains, transmission to hands of hospital personnel, and its prevention by use of a new heating device. Zentralbl Hyg Umweltmed.

[3] Kramer A, Daeschlein G, Niesytto C, Sissoko B, Sütterlin R, Blaschke M, Fusch C. Contamination of sinks and emission of nosocomial gramnegative pathogens in a NICU - outing of a reservoir as risk factor for nosocomial colonization and infection. Umweltmed. Forsch. Prax. 2005;10(5):327.

[4] StarlanderMelhus Å G, Melhus Å (2012). Minor outbreak of extended-spectrum β-lactamase-producing Klebsiella pneumoniae in an intensive care unit due to a contaminated sink. J Hosp Infect.

[5] Lowe C, Willey B, O’Shaughnessy A, Lee W, Lum M, Pike K, Larocque C, Dedier H, Dales L, Moore C (2012). Outbreak of extended-spectrum β-lactamase-producing Klebsiella oxytoca infections associated with contaminated handwashing sinks. Emerg Infect Dis.

[6] Wolf I, Bergervoet P, Sebens F, van den Oever H, Savelkoul P, van der Zwet W (2014). The sink as a correctable source of extended-spectrum β-lactamase contamination for patients in the intensive care unit. J Hosp Infect.

[7] Roux D, Aubier B, Cochard H, Quentin R, van der Mee-Marquet N (2013). Contaminated sinks in intensive care units: an underestimated source of extended-spectrum beta-lactamase-producing Enterobacteriaceae in the patient environment. J Hosp Infect.

[8] Leitner E, Zarfel G, Luxner J, Herzog K, Pekard-Amenitsch S, Hoenigl M, Valentin T, Feierl G, Grisold A, Högenauer C, Sill H, Krause R, Zollner-Schwetzd I (2015). Contaminated Handwashing Sinks as the Source of a Clonal Outbreak of KPC-2-Producing Klebsiella oxytoca on a Hematology Ward. AAC.

[9] Hoxha A, Kärki T, Giambi C, Montano C, Sisto A, Bella A, D’Ancona F (2016). Attributable mortality of carbapenem-resistant Klebsiella pneumoniae infections in a prospective matched cohort study in Italy, 2012–2013. J Hosp Infect.

[10] Wilson AP, Livermore DM, Otter JA, Warren RE, Jenks P, Enoch DA, Newsholme W, Oppenheim B, Leanord A, McNulty C, Tanner G, Bennett S, Cann M, Bostock J, Collins E, Peckitt S, Ritchie L, Fry C, Hawkey P (2016). Prevention and control of multi-drug-resistant Gram-negative bacteria: recommendations from a Joint Working Party. J Hosp Infect.

[11] Hota S, Hirji Z, Stockton K, Lemieux C, Dedier H, Wolfaardt G, Gardam MA (2009). Outbreak of multidrug-resistant Pseudomonas aeruginosa colonization and infection secondary to imperfect intensive care unit room design. Infect Control Hosp Epidemiol.

[12] Sissoko B, Sütterlin R, Blaschke M, Flicker J, Schluttig A (2004). Infektionreservoir Geruchsverschluss: Prävention nosokomialer Infektionen. Hyg Med.

